# Kinetic Study of Oxygen Adsorption over Nanosized Au/γ-Al_2_O_3_ Supported Catalysts under Selective CO Oxidation Conditions

**DOI:** 10.3390/molecules17054878

**Published:** 2012-04-27

**Authors:** Dimitrios Gavril, Aglaia Georgaka, George Karaiskakis

**Affiliations:** 1 Physical Chemistry Laboratory, Department of Chemistry, University of Patras, Patras 26504, Greece; Email: AGeorgaka@eap.gr (A.G.); G.Karaiskakis@chemistry.upatras.gr (G.K.); 2 School of Science and Technology, Hellenic Open University, Patras 26223, Greece

**Keywords:** nanoparticles, Au/γ-Αl_2_Ο_3_, catalysts, SCO, PROX, reversed-flow inverse gas chromatography, inverse gas chromatography, rate constants, oxygen, carbon monoxide

## Abstract

O_2_ adsorption is a key process for further understanding the mechanism of selective CO oxidation (SCO) on gold catalysts. Rate constants related to the elementary steps of O_2_ adsorption, desorption and surface bonding, as well as the respective activation energies, over a nanosized Au/γ-Al_2_O_3_ catalyst, were determined by Reversed-Flow Inverse Gas Chromatography (RF-IGC). The present study, carried-out in a wide temperature range (50–300 °C), both in excess as well as in the absence of H_2_, resulted in mechanistic insights and kinetic as well as energetic comparisons, on the sorption processes of SCO reactants. In the absence of H_2_, the rate of O_2_ binding, over Au/γ-Al_2_O_3_, drastically changes with rising temperature, indicating possible O_2_ dissociation at elevated temperatures. H_2_ facilitates stronger O_2_ bonding at higher temperatures, while low temperature binding remains practically unaffected. The lower energy barriers observed, under H_2_ rich conditions, can be correlated to O_2_ dissociation after hydrogenation. Although, H_2_ enhances both selective CO reactant’s desorption, O_2_ desorption is more favored than that of CO, in agreement with the well-known mild bonding of SCO reactant’s at lower temperatures. The experimentally observed drastic change in the strength of CO and O_2_ binding is consistent both with well-known high activity of SCO at ambient temperatures, as well as with the loss of selectivity at higher temperatures.

## 1. Introduction

The discovery of the catalytic properties of gold nano-particles was one of the most exciting recent scientific findings, which led to a true renaissance of gold chemistry, and catalysis by gold has rapidly become a hot topic in chemistry. Catalytic applications of gold have been the subject of several review articles [1,2,3,4,5,6,7,8,9,10,11,12,13,14,15,16,17,18,19,20,21,22,23,24,25,26], and collections of papers have been edited in journal special issues, over the last two decades.

Catalytic applications of Au concern many important processes, of significant technological and environmental interest, such as: low-temperature CO oxidation [3,6,25,27,28,29,30,31,32,33,34,35,36,37,38,39,40], water gas shift reactions [41,42], C–C coupling reactions [43], synthesis of N- and O-heterocycles [44] and hydrogenation of organic compounds [45].

In the 1980s, Haruta *et al*. recognized that supported gold nanocrystals can be highly effective catalysts for the oxidation of CO at temperatures considerably below 0 °C. The high activity of supported gold catalysts for the oxidation of CO at ambient temperature should be primarily of interest for respiratory protection. However, the particular application for which supported gold catalysts have been studied profusely is the purification of the H_2_ streams in fuel cells [14,46], and in particular in polymer electrolyte membrane fuel cells (PEMFCs), which are used in electric vehicles and operate at about 80–100 °C. Gold catalysts are also candidates for the production of H_2_ by steam reforming of methanol [47].

PEMFCs operation is based on the oxidation of H_2_ produced from methanol by steam reforming and water gas shift reactions. Residual CO can poison the Pt anode at the low operating temperature, and hence trace amounts of CO have to be removed from the H_2_ stream in the presence of water to ensure long cell lifetimes. Although conceptually simple, the oxidation of CO in the presence of excess moist H_2_, without oxidizing the H_2_, is a particularly difficult reaction which has attracted considerable research efforts. Thus, an efficient selective CO oxidation (SCO) catalyst must be highly active in CO oxidation, at temperatures compatible with the operation of PEM fuel cells, and very selective towards CO_2_ formation.

Nanometer-sized gold particles supported on γ-Al_2_O_3_ have been investigated as efficient catalysts for the low temperature CO oxidation as well as the selective catalytic oxidation of CO, under conditions compatible with the operation of PEM fuel cells [48]. Among the various supported Au catalysts, Au/Al_2_O_3_ is perhaps one that has shown the widest variation for CO oxidation, ranging from being very inactive to highly active. In the present work, a well-studied efficient catalyst consisting of Au nanoparticles supported on an inert material, such as γ-Al_2_O_3_, has been utilized as model system due to its simplicity, in order to achieve physicochemical insights on the catalytic behaviour of gold nanoparticles.

It is generally agreed that the exceptional catalytic activity of gold depends on the size of the gold particles, but the nature of support material, the preparation method, and/or the activation procedure have also all been suggested to play a key role [3]. Sites at the gold support interface have also been claimed to be responsible for the activity in CO oxidation [49]. Strain in the Au particles due to the mismatch of the lattices at the interface with the support and the effect of low-coordinated sites and roughness have also been suggested as important factors for high activity [50]. The availability of many low-coordinated gold atoms on the small particles is considered as the most important effect, concerning the catalytic activity of gold nanoparticles for the low temperature oxidation of CO [33]. Effects related to the interaction with the support may also contribute, but to a considerably smaller extent [33].

For successful operation as a selective CO oxidation catalyst in a reformer-PEMFC system, the catalyst must operate in a complex feed comprising CO, O_2_, H_2_, CO_2_, H_2_O, and N_2_, and be capable of converting almost 100% of the CO. Taken together, this represents a demanding target, in which coadsorption effects play a vital role in the catalytic action, since they have a large effect on the kinetics and dynamics of surface reactions.

Although CO oxidation over Group 10 noble metals is the most studied catalytic reaction, selective oxidation of CO under H_2_-rich conditions is a topic of significant recent study [51,52]. Due to the high adsorption energy of CO, for platinum-based catalysts the active metal is shielded by a dense CO adlayer under typical reaction conditions, and the presence of other adsorbates has rather small, “indirect” effects, caused by adsorption on the support. In contrast, on supported gold catalysts the rather low CO adsorption energy allows significant coadsorption on the metal surface, which in turn may result in much more severe effects [53]. Kinetic effects of O_2_ on the CO oxidation reaction over gold have rarely been studied experimentally.

This is the novelty of the present study. O_2_ adsorption is a key process for further understanding the mechanism of selective CO oxidation on gold catalysts. The effects of H_2_ and temperature, on the rates related to the elementary steps of O_2_ adsorption (*k*_1_), desorption (*k*_−1_) and surface bonding (*k*_2_), over the studied Au/γ-Al_2_O_3_ catalyst, were investigated by Reversed-Flow Inverse Gas Chromatography (RF-IGC). The present study was carried-out in a wide temperature range (50–300 °C), both in excess as well as in the absence of H_2_, permitting mechanistic insights and kinetic as well as energetic comparisons, of the sorption processes of SCO reactants over Au/γ-Al_2_O_3_.

## 2. Results and Discussion

### 2.1. Effects of Temperature and H_2_ in O_2_ Sorption

**Figure 1:**
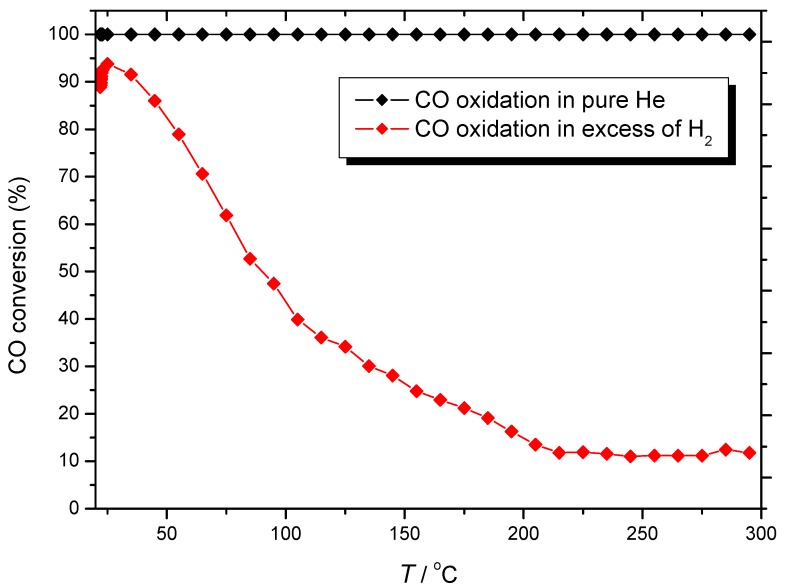
Temperature variation of CO to CO_2_ conversion, measured by TPSR, over the studied Au/γ-Al_2_O_3_ catalyst, both in the absence as well as in excess of H_2_. Symbols: (black♦) CO/O_2_ = 1:1 in He, and (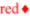) CO/O_2_ = 1:1 in a H_2_-rich environment.

The performance of the Au/γ-Al_2_O_3_ catalyst both under normal preferential oxidation (PROX)-type operation as well as for normal CO oxidation has been studied in detail in previous publications [48,53,54,55]. The findings of these studies are summarized in Figure 1, in which temperature variation plots of CO to CO_2_ conversion, measured by the temperature programmed surface reaction (TPSR) technique, over the studied Au/γ-Al_2_O_3_ catalyst both in the absence as well as in excess of H_2_, are shown. It was observed that in the absence of H_2_ CO is totally oxidized within the whole studied temperature range. In contrast, in excess of H_2_, CO conversion to CO_2_ is high and selective at lower temperatures, while at higher temperatures a drastic decrease of CO conversion is noticed, which is further accompanied by loss of selectivity towards water formation. It is well-known that Au/γ-Al_2_O_3_ catalysts are not very good at temperatures compatible with PEM fuel cells (80–100 °C). However, Au supported on an inert material such as γ-Al_2_O_3_ is a good model system for studying SCO reactants sorption processes over nanosized Au.

The effects of H_2_ and CO_2_ on the rates related to the elementary steps of CO sorption over Au/γ-Al_2_O_3_ have also been studied [54]. The experimental findings obtained both by TPSR, as well as by RF-IGC, indicated the “decomposition” of CO to CO_2_ on the studied catalyst, in the absence of H_2_. The explanation of CO decomposition was based on a model of active sites consisting of an ensemble of metallic Au atoms and a cationic Au with a hydroxyl group. These Au^+^-OH^-^ groups contribute to the low temperature oxidation of CO in the absence of H_2_, but it is not expected to contribute to CO selective oxidation in excess of H_2_.

A significant experimental finding was the reverse water-gas shift (RWGS) reaction of CO_2_ to CO, which further contributes to the well-known drastic decrease of SCO activity and selectivity, at high temperatures. Furthermore, it was found that carbon dioxide adsorption competes that of CO, under H_2_-rich conditions. However, the strength of CO_2_ bonding was higher compared to that of CO and it further increased at higher temperatures. The stronger CO_2_ bonding was explained on the basis that it takes place much more likely at active sites located on the γ-Al_2_O_3_ support.

O_2_ is the other reactant of selective CO oxidation. The experimental rates for O_2_ adsorption (*k*_1_), desorption (*k*_−1_), and surface bonding (*k*_2_), at various temperatures, over the studied Au/γ-Al_2_O_3_ catalyst, are given in Table 1, both in the absence as well as in excess of H_2_. The statistical analysis of O_2_ rate constants, based on the average uncertainty of these values (5.8% for *k*_1_, 4.5% for *k*_−1_ and 7.5% for *k*_2_, respectively) ascertains that the experimental rates determined by RF-IGC can lead to safe conclusions.

**Table 1:** Experimentally determined rate constants, for O_2_ adsorption (*k*_1_), desorption (*k*_−1_), and surface bonding (*k*_2_), over Au/γ-Al_2_O_3_ catalyst, at various temperatures, both in the absence as well as in excess of H_2_ in the carrier gas.

% H_2_	*T*/°C	*k*_1_/s^−1^	% error *	10^3^ *k*_−1_/s^−1^	% error *	10^3^ *k*_2_/s^−1^	% error *
	50	0.09		0.69		0.17	
	70	0.11		0.68		0.19	
	100	0.16		0.73		0.20	
0	150	0.12		1.09		0.23	
	200	0.10		1.44		0.26	
	250	0.19		1.53		0.51	
	300	0.18		1.10		1.18	
	30	0.17 ± 0.006	3.5	0.84 ± 0.022	2.6	0.21 ± 0.036	17
	50	0.18 ± 0.003	1.7	1.30 ± 0.017	1.3	0.22 ± 0.017	7.7
	70	0.24 ± 0.023	9.6	1.59 ± 0.024	1.5	0.23 ± 0.025	11
	90	0.27 ± 0.012	4.4	1.75 ± 0.036	2.1	0.25 ± 0.013	5.2
75	100	0.27 ± 0.008	3.0	1.76 ± 0.044	2.5	0.27 ± 0.020	7.4
	150	0.22 ± 0.027	12	2.33 ± 0.236	10	0.39 ± 0.031	7.9
	200	0.33 ± 0.010	3.0	3.00 ± 0.231	7.7	0.56 ± 0.031	5.5
	250	0.35 ± 0.025	7.1	2.98 ± 0.215	7.2	0.74 ± 0.009	1.2
	300	0.49 ± 0.035	7.7	3.52 ± 0.206	5.9	0.91 ± 0.038	4.2
Average uncertainty %			5.8		4.5		7.5

* error (%) expressing the uncertainty of the kinetic measurements, is defined as the ratio of the standard deviation divided by the average value of the measured rate constants.

**Scheme 1:**
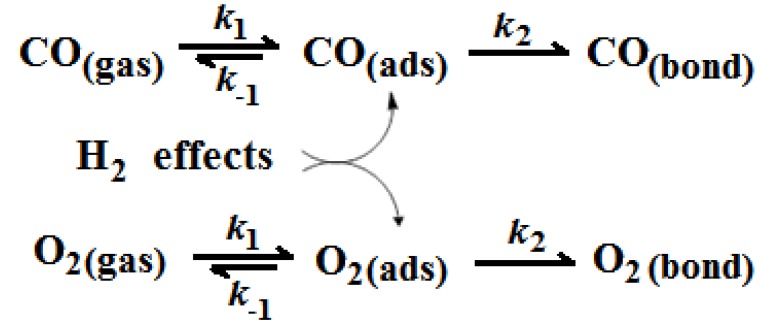
Mechanistic scheme of the effect of H_2_ in CO and O_2_ sorption, over Au/γ-Αl_2_O_3_ catalyst.

Comparing the values of the respective sorption rates, information concerning the mechanism of SCO reactants sorption over Au/γ-Al_2_O_3_ catalyst can be extracted. It is observed that the rates of adsorption and desorption are higher than those of surface binding. Moreover adsorption rate is higher than that of desorption. These findings suggest the mechanism shown in Scheme 1, in which the faster steps of adsorption and desorption are followed by the slower step of surface bonding.

It becomes clear that the experimentally determined O_2_ sorption rates increase with rising temperature, as it is expected for activated processes. Moreover, in all cases *k*_1_ rates are higher than *k*_−1_, which are also higher than *k*_2_ ones. However, it is also noticeable that in the absence of H_2_, at temperatures higher than 200 °C, the rate of O_2_ surface bonding drastically increases, and at 300 °C its value becomes almost the same as the respective desorption rate.

The question naturally arising is how H_2_ affects the rate of each process related to O_2_ sorption on Au/γ-Al_2_O_3_. It is concluded, from the higher rates, that H_2_ plays a beneficial role in both the processes of O_2_ adsorption and desorption, over the studied Au/γ-Al_2_O_3_ catalyst. The rate of O_2_ surface bonding is also enhanced in almost the whole studied temperature range. However, it is also clear that for the process of O_2_ surface bonding in the absence of H_2_, there is a “turning point” temperature. While at lower temperatures O_2_ bonding increases slightly, at temperatures higher than 200 °C, the rate of O_2_ surface bonding drastically increases and at 300 °C its value becomes higher that in excess of H_2_. Moreover, we can notice a similar change in O_2_ desorption from Au/γ-Al_2_O_3_ catalyst, when H_2_ is not present in the carrier gas, at temperatures higher than 200 °C. At these temperatures the rate of O_2_ desorption first stabilizes and at temperatures higher than 250 °C decreases.

Bearing in mind the above observations, the respective activation energies were calculated for the adsorption, desorption as well as surface binding of O_2_ on the studied Au catalyst, by means of Arrhenius equation, by plotting ln(*k*) *vs*. 1/*T*. However, for O_2_ surface bonding two activation energies were extracted: one at lower temperatures and another corresponding at temperatures higher than 200 °C, in the case where H_2_ is absent and higher than 100 °C for conditions in excess of H_2_. 

**Figure 2:**
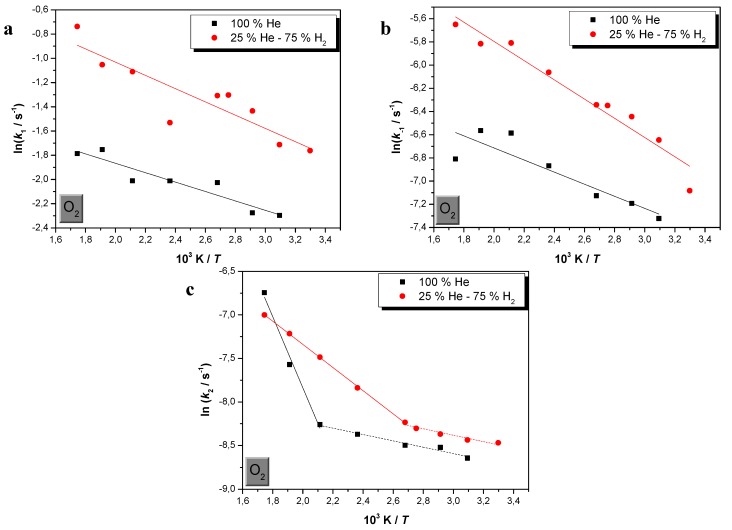
Comparative Arrhenius plots exhibiting the effects of H_2_ and temperature in O_2_ adsorption (**a**), desorption (**b**) and surface bonding (**c**), over Au/γ-Al_2_O_3_, in excess and absence of H_2_, within the carrier gas.

Comparative Arrhenius plots showing the effect of H_2_ in the respective sorption processes over Au catalyst are given in Figure 2. The mean values of the activation energies as well as their standard errors and the correlation coefficients, estimated for all the activated processes, in the absence and in excess of H_2_, are compiled on Table 2.

**Table 2:** Mean activation energy values, their standard error and their correlation coefficient *r* (in brackets), calculated by means of Arrhenius equation, for O_2_ adsorption, desorption and surface bonding, over the studied nanosized Au/γ-Al_2_O_3_ catalyst, in excess as well as in the absence of H_2_, within the carrier gas.

% H_2_	*Ε*a_1_/kJ·mol^−1^	*Ε*a_−1_/kJ·mol^−1^	*Ε*a_2_/kJ·mol^−1^	
0	3.2 ± 0.5 (0.864)	4.3 ± 1.0 (0.765)	3.0 ± 0.3 * (0.961)	34.0 ±3.6 * (0.977)
75	4.6 ± 0.8 (0.781)	6.9 ± 0.6 (0.942)	3.1 ± 0.5 ** (0.917)	11.1 ± 0.1 ** (0.999)

* Surface bonding activation energy at *T *< 100 and >100 °C, respectively; ** Surface bonding activation energy at *T *< 200 and >200 °C, respectively.

The normal question is how the present kinetic findings compared with the literature data. In order to obtain a comprehensive picture of CO oxidation on Au surfaces DFT calculations and experimental methods have been used and many possible elementary reactions in CO oxidation on a range of Au surfaces have been calculated [27,28,29,30,31,32,33,34,35,36,37,38,39,40,41,42,49,50,56]. It is expected that O_2_ cannot dissociate at low temperatures, but it may be possible on steps at elevated temperatures. The barriers of O_2_ dissociation on Au surfaces are generally very high and the lowest barrier for O_2_ dissociation is 0.93 eV (89.4 kJ·mol^−1^), which occurs on Au steps [27].

In a recent work the mechanism and kinetics of CO oxidation in SCO over Au surfaces have been investigated using DFT [28]. These authors found that direct bi-molecular reactions between O_2_ and H_2_ cannot take place because of the high activation barrier of 2.05 eV (197.1 kJ·mol^−1^) and concluded that H_2_ dissociation is required for the SCO reaction. Au systems containing low coordinated Au atoms are capable of dissociating H_2_ below room temperature. Extending the general finding that O_2_ cannot dissociate at low temperatures, they have concluded that O_2_ dissociation after hydrogenation occurs easily and the CO oxidation rate is enhanced. O_2_ hydrogenation strongly enhances the surface-O_2_ interaction and assists in scission of the O–O bond. Thus, the activation energy required to make the reaction intermediate hydroperoxy (OOH) from O_2_ and H is small. They have calculated O_2_ adsorption energies of −0.12, −0.52 and −0.17 eV, (11.5, 50.0 and 16.3 kJ·mol^−1^) respectively on the (100), the diatomic rows on (100) and the (310) Au surfaces.

In another theoretical work, DFT studies of the reactivity towards CO oxidation of supported on oxides Au nanoparticles were reviewed [56]. The calculated adsorption potential energies for O_2_ bonded to a supported Au_34_ particle vary from 0.03 to 0.40 eV (2.9–38.5 kJ·mol^−1^), depending on the configuration. The binding of molecular O_2_ is very weak and rather insensitive to the environment. They noticed that the weak molecular O_2_ chemisorption at low temperatures is found to be much smaller for Au(111) single crystals than for small TiO_2_-supported Au particles. They also noted, indications of a moderate O_2_-gold binding for small Au clusters [29] or highly undercoordinated Au atoms [Au adatom on Au(111)].

From the above presented literature survey, concerning O_2_ adsorption energies either due to adsorption on clean Au surfaces or supported Au nanoparticles, it is clear that the values of the experimentally determined activation energies lie within the expected binding energy range, varying from 3.0 to 34.0 kJ·mol^−1^. These values indicate that O_2_ adsorption, desorption and low temperature surface binding are weak. The found low activation energy values are in agreement with the well-expected weak binding of molecular O_2_ on Au, which is insensitive to the environment [56].

However, the values of the activation energies, corresponding to each sorption step of adsorption, desorption and low temperature surface bonding, are slightly different. Within the range of the experimental error, the activation energies corresponding to O_2_ desorption are slightly higher than those of adsorption, which in their turn are slightly higher than those due to surface bonding, at lower temperatures. Moreover, it is observed that H_2_ slightly affects the adsorption/desorption activation energies, from which only that of desorption slightly increases, under H_2_-rich conditions.

The values of the activation energies indicate that O_2_ binding over Au/γ-Al_2_O_3_ catalyst is weaker at lower temperatures and it becomes stronger at higher temperatures. This finding makes clear that there is a noticeable difference concerning the nature of O_2_ binding over Au/γ-Al_2_O_3_, at lower and higher temperatures. Such a behavior is in agreement with the general assumption that O_2_ cannot dissociate at low temperatures, but it may be possible on steps at elevated temperatures [27,28,56].

Moreover, H_2_ affects to a higher extent O_2_ binding at elevated temperatures, compared to adsorption, desorption and binding at lower temperatures. In this case, H_2_ facilitates strong O_2_ bonding (from 34.0 to 11.1 kJ·mol^−1^) while low temperature binding remains practically unaffected. This experimentally observed behavior is also consistent with the well-known SCO loss of selectivity at elevated temperatures [27].

The lower energy barriers observed, under H_2_ rich conditions, can be correlated with the finding of a recent DFT study, that although O_2_ cannot dissociate at low temperatures, O_2_ dissociation after hydrogenation occurs easily and the CO oxidation rate is enhanced. In the same work, it is suggested that hydroperoxy intermediates may migrate from the oxide support to Au particles. The hydroperoxy intermediates on the support is formed as a consequence of reaction of O_2_ with H (spilled over from Au) [28].

### 2.2. Comparison of CO and O_2_ Sorption

**Figure 3:**
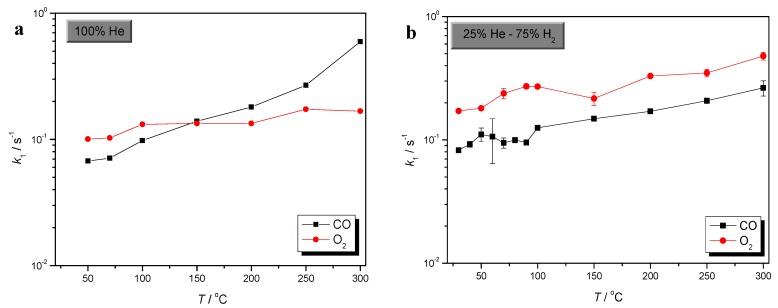
Comparative plots of CO and O_2_ adsorption rate, in the absence (**a**), as well as in excess of H_2_ (**b**).

**Figure 4:**
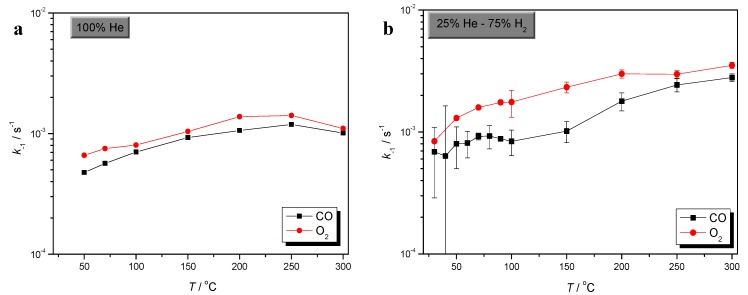
Comparative plots of CO and O_2_ desorption rate, in the absence (**a**), as well as in excess of H_2_ (**b**).

**Figure 5:**
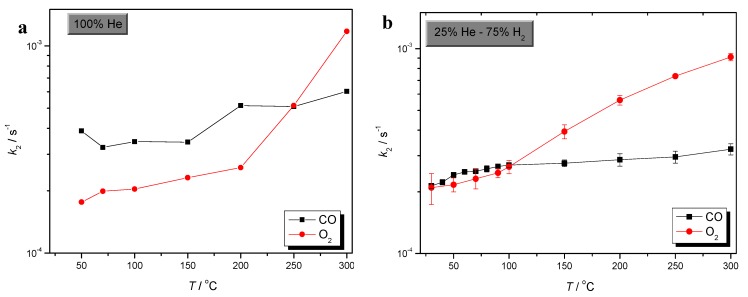
Comparative plots of CO and O_2_ surface binding rate, in the absence (**a**), as well as in excess of H_2_ (**b**).

Comparison of CO and O_2_ sorption processes could give very useful information concerning selective CO oxidation over Au/γ-Al_2_O_3_. Comparative plots of CO and O_2_ sorption rates, in the absence of H_2_, as well as in excess of H_2_, are presented in Figure 3, Figure 4 and Figure 5, respectively.

From the plots of Figure 3, it is observed that in the absence of H_2_, the rate of O_2_ adsorption is slightly higher than that of CO below 150 °C , at 150 °C becomes equal and above 150 °C this trend is reversed and CO adsorption rate becomes higher.

In excess of H_2_, the rate of O_2_ adsorption is higher than that of CO, in the entire temperature range. It is generally observed that both the processes of CO and O_2_ adsorption are enhanced by the presence of H_2_. However, the drastic increase of the rate of CO adsorption, which is observed at higher temperatures and in the absence of H_2_, should be noted.

Conclusions, concerning the rate of desorption of both SCO reactants, can be drawn from the plots of Figure 4. O_2_ desorption is slightly higher than that of CO, in the entire temperature range, both in the presence as well as in the absence of H_2_. Moreover, H_2_ enhances both reactant’s desorption. However, it is also clear that O_2_ desorption is more favored by that of CO. These findings are in agreement with the well-known mild bonding of SCO reactants at lower temperatures [27,28,56].

In addition, it is also noticeable that in the absence of H_2_ and at *T *> 200 °C the rates of CO as well as O_2_ desorption from Au/γ-Al_2_O_3_ first stabilize, within the range from 200 to 250 °C, and at even higher temperatures decrease. This behavior can be related to the well-expected stronger binding of both SCO reactants at high temperatures [27,28,56].

The strength of the adsorbate-Au/γ-Al_2_O_3_ interaction is indicated by the variation of *k*_2_ values, shown in Figure 5. In the absence of H_2_, O_2_ binding is clearly lower compared to that of CO. However, at elevated temperatures the rate of O_2_ binding drastically increases, and at *T* > 250 °C surpasses that of CO.

Although, under H_2_ rich conditions, the rates of both CO and O_2_ binding over Au/γ-Al_2_O_3_ are almost equal at lower temperatures, at temperatures higher than 100 °C the strength of O_2_ binding becomes higher and increases drastically as temperature elevates. Moreover, it is observed that the binding of both SCO reactants becomes weaker at the low temperature range, in agreement with the findings of theoretical studies, which summarize the role of H_2_ that the weakening of the strength of the CO and O_2_ bonding, due to H_2_, intensifies the possibility of CO+O_2_ reaction on Au [26,28,29,35]. This drastic change in the strength of CO and O_2_ binding on Au/γ-Al_2_O_3_ catalyst is consistent both with well-known high activity of SCO at ambient temperatures as well as with the loss of selectivity, which is observed at higher temperatures [27,28,56].

These findings confirm various aspects of the low temperature CO oxidation mechanism. Possible O_2_ dissociation (O_2_→2O_ad_) on Au cannot occur at low temperatures, as it is indicated by the high activation O_2_ binding energy of 34.0 kJ·mol^−1^ of this work, and it is expected that CO oxidation on Au/inactive-materials occurs on Au steps via a two-step mechanism: a slow CO + O_2_→ CO_2_ + O reaction, and then a fast CO + O → CO_2_ reaction [20,27].

The same mechanism generally describes selective CO oxidation in excess of H_2_.On the other hand, H_2_ plays an important role facilitating the activation of O_2_ molecules (e.g., through hydroperoxy intermediates species) or with O_2_ bound to the support [26,28,29,35].

## 3. Experimental

### 3.1. Preparation of Nanometer Size Au Catalysts

The nanometer sized Au/γ-Al_2_O_3_ catalyst with an intended 5% wt. composition was prepared by homogeneous deposition precipitation. This method leads to the smallest average gold size (3–5 nm). Αn aqueous solution of hydrogen tetrachloroaurate(III) (HAuCl_4_) and excess urea was added to the support (pH 4). The suspension was then vigorously stirred and heated to 70–75 °C to ensure a slow decomposition of the urea. The formation of CO_2_ drives the reaction completely to the right. Because OH^-^ is also being formed the pH of the solution increases gradually with advancing decomposition. Since the pH is controlled by a chemical reaction, which takes place throughout the whole solution, a more homogeneous increase of the pH is achieved than when directly injecting a base. After the pH reached the value of 8, the slurry was filtered, washed to remove Cl^−^ and dried at ambient conditions.

Finally, gold catalyst was dried in air at 80 °C for at least 16 h and calcined in a flow of O_2_ up to 300 °C (heating rate 5 °C·min^−1^). The sample was kept at 300 °C for 2 h, and then cooled to room temperature. Before any use, the catalyst was reduced at 300 °C for 10 h in flowing H_2_.

The method of preparation and the surface characterization of the catalyst using AAS, XRD, HRTEM, XPS and MES, have been presented previously [48,53]. The gold loading determined by AAS was 5.1 ± 0.3%, while the mean diameter of the gold particles was 4.2 nm (XRD) and 3.6 ± 1.4 nm (HRTEM), respectively.

### 3.2. Materials

H_2_, from Linde (Patras, Greece, 99.999% pure) was used for the reduction of the catalyst. Pure He and a mixture consisting of 25% He + 75% H_2_, from BOC Gases (Athens, Greece, 99.999% pure) were used as carrier gases. O_2_ was from BOC Gases (Athens, Greece, 99.997% pure).

### 3.3. Kinetic Measurements

**Figure 6:**
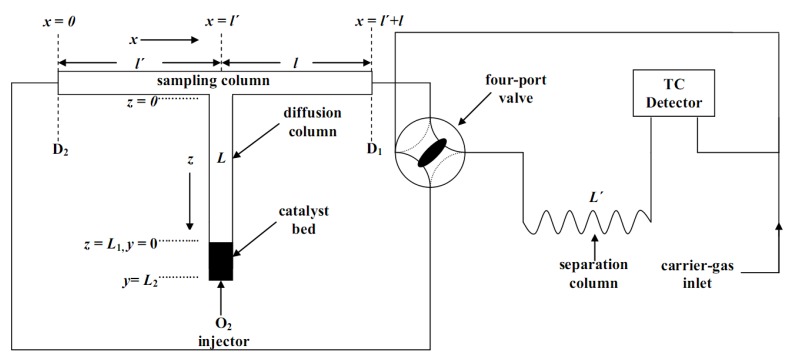
Experimental setup of Reversed-Flow Inverse Gas Chromatography used for the kinetic study of O_2_ adsorption over Au/γ-Al_2_O_3_ catalysts.

Kinetic measurements were performed by using the gas chromatographic technique of reversed-flow inverse gas chromatography. In RF-IGC, another column (diffusion column) is placed perpendicularly in the centre of the usual chromatographic column (sampling column), as shown in Figure 6. The carrier gas flows continuously through the sampling column, while it is stagnant inside the diffusion column. RF-IGC can be considered as an inverse gas chromatographic method, since in RF-IGC the solid or liquid substance placed into the diffusion column is under investigation. The catalyst bed is placed at the lower closed end of the diffusion column. The displacement of the injected solute into the diffusion column depends on its interaction with the stationary phase as well as its diffusion into the stagnant carrier gas (mobile phase), while it is independent of the carrier gas flow-rate making RF-IGC an attractive method for physicochemical measurements. The estimation of the various parameters is done from plots of the heights or the areas of the extra chromatographic peaks against the time from solute’s injection and from geometrical characteristics of the diffusion column (such as its length and its volume).

RF-IGC has been used successfully for the determination of diffusion coefficients in binary and ternary gas mixtures [57,58], Lennard-Jones parameters of gases [59], mass transfer coefficients on liquids *etc*. [60,61,62,63]. During the last decade, a large number of physicochemical quantities related to gas-solid interactions have also been determined by RF-IGC methodologies [64,65,66,67,68,69,70]. The determination of these quantities gives important information for the mechanism of the respective processes and permits the characterization of the studied solids. Before utilizing RF-IGC methodologies in the study SCO over gold catalysts, they have been validated by studying the sorption processes of CO, O_2_ and CO_2_ over well-studied silica supported Pt, Rh and Pt-Rh bimetallic catalysts [67,68,69,70].

#### 3.3.1. Apparatus and Procedure

The lengths 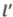, *Ɩ* and L of the stainless-steel “sampling cell” were incorporated into a commercial gas chromatograph, Shimadzu GC-8A (Tokyo, Japan), equipped with a thermal conductivity detector as shown in Figure 6. The lengths 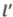 and *Ɩ* of the sampling column were 36.5 cm each (4 mm ID), while the length *L* of the diffusion column was 70 cm (4 mm ID). The catalytic bed (0.09 g) was put in a 1 cm length at the closed end of the diffusion column *L*. The separation column, 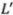 (4 mm ID), was filled with 7.6 g of silica gel (80–100 mesh).

After catalyst reduction, the whole system was conditioned by heating *“in situ”* the catalyst bed at 748 K for 20 h, under a continuous carrier gas flow. Some preliminary injections of O_2_ were made to stabilize the adsorptive behavior. Then, 1.0 cm^3^ of the solute, under atmospheric pressure was rapidly introduced, with a gas-tight syringe, at the top of the diffusion column *L*, with the carrier gas flowing in direction from *D_1_* to *D_2_* (*c.f*. Figure 6). After a time of 5 min, a continuous concentration-time curve, owing to the adsorbate was established and recorded. Flow reversals of carrier gas direction, for 5 s, were made and then the gas was again turned to its original direction, simply by switching the four-port valve from one position to the other and *vice-versa*. This time period was shorter than the gas hold-up time in column sections 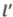, *Ɩ* and 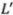. When the gas flow was restored to its original direction sample peaks, similar to those of Figure 2 of Ref. [67], were recorded. Repeating the above reversal procedure many times at each temperature, a whole series of sample peaks were recorded, corresponding to a different time from adsorbate injection. The working temperature for the chromatographic material was kept constant at 85 °C. The variation in the temperature along the catalytic bed was measured by a Fluke 2190A digital thermometer and was smaller than 1 K. The volumetric carrier gas flow rate, at ambient temperature was 1.136 cm^3^s^−1^. The pressure drop along the whole system was 0.33 atm. Plots of the height of the sample peaks against the corresponding time from adsorbate’s injection produce the so-called *diffusion band*”, like those shown in Figure 3 of ref. [67].

#### 3.3.2. Theoretical

The sampling peaks are predicted theoretically by the “chromatographic sampling equation” describing the concentration-time curve recorded after each flow reversal. The area, *A*, or the height, *H*, of the sample peaks resulting from the flow reversals, measured as function of time, *t*, are proportional to the concentration of the substance under study, at the junction, 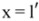, of the sampling cell, at time, *t*:


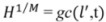
(1)

where *M* is the response factor of the detector and *g* a proportionality constant (usually assumed equal to unity) characteristic of the detector. Measuring experimentally pairs *H*, *t*, the diffusion band is constructed, which in the case of a solute adsorption is theoretically described by the sum of two exponential functions of time [67]:


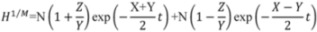
(2)

*N* is a function of the amount of the injected adsorbate, of the carrier gas volumetric flow-rate, of the geometrical characteristics of the diffusion column and of the diffusivity of the adsorbate into the carrier gas. Its value, included in the pre-exponential factors of Equation (2), is eliminated during the calculations.

The pre-exponential factors, *A*_1_, *A*_2_ and the exponential coefficients of time 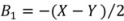 and 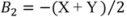, are calculated by a non-linear regression analysis program. The auxiliary parameters *X*, *Y* and *Z* are functions of the rate constants *k*_1_, *k*_−1_ and *k*_2_ as well as of the geometric characteristics of the diffusion column and the diffusivity of the adsorbate into the carrier gas. By adding the two exponential coefficients of time *B*_1_ and *B*_2_, the value of *X* is found, while subtracting *B*_1_ and *B*_2_, the value of *Y* is obtained. The value of *Z* is found from the ratio of the two pre-exponential factors *A*_1_ and *A*_2_ (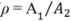):


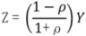
(3)

Finally, the rate constants for adsorption, *k*_1_, desorption, *k*_−1_ and surface reaction, *k*_2_ can be calculated from the found values of *X*, *Y* and *Z*, by means of the following relations [67]:


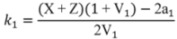
(4)


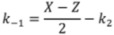
(5)


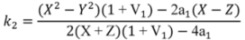
(6)


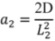
(7)


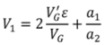
(8)

In the above equations, *V*_G_ and 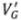 are the gaseous volumes of empty sections *L*_1_ and *L*_2_, respectively, a_1_, a_2_ functions of the diffusion coefficient, *D*, of the adsorbate at the experimental temperature and pressure and *ε *is the external porosity of catalyst bed.

Equations (4)–(6) show that for the calculation of the rate constants *k*_1_, *k*_−1_ and *k*_2_, the following parameters are required: (i) the auxiliary parameters *X*, *Y* and *Z* determined by the pre-exponential factors and exponential coefficients of Equation (2), which are easily calculated by nonlinear regression analysis of the diffusion band; (ii) the diffusion coefficient of the adsorbate, *D*. The determination of diffusion coefficients can be done either experimentally [57] or theoretically; (iii) the parameter *V*_1_ defined by Equation (8), is easily calculated by the geometric properties of the diffusion column and the value of the diffusion coefficient of O_2_, *D*. (iv) The external porosity of the catalyst bed, *ε*, which can be measured by a method described elsewhere [67].

#### 3.3.3. Precision Analysis of Rate Constants Determination by RF-IGC

The interaction of each of SCO desirable reactants (CO, O_2_) and product (CO_2_) over the studied catalyst can be approached by a scheme in which the elementary reversible steps of adsorption, *k*_1_, and desorption, *k*_−1_, are followed by their possible surface bonding, *k*_2_. It is generally difficult to estimate the errors of the rate constants, emerged from a long sequence of steps of rather complex calculations, at which the application of the rule of error propagation does not give reliable final errors. For this reason, a preliminary precision analysis was carried-out, by performing ten consecutive experiments, utilizing CO sorption in excess of H_2_, at 100 °C.

The found precision of the experimentally determined rates is satisfactory: *k*_1_ = (8.7 ± 0.3)∙10^−2^·s^−1^ (3.5%), *k*_−1_ = (11.1 ± 0.2)∙10^−4^·s^−1^ (2.0%), *k*_2_ = (1.7 ± 0.4)∙10^−4^·s^−1^ (2.6%). Thus, the average precision of the method was found: 96.5% for *k*_1_, 98.0% for *k*_−1_ and 97.4% for *k*_2_, respectively. These findings ascertain that the so determined rates can lead to safe conclusions. Moreover the rates corresponding to the elementary sorption processes over the studied catalyst (in excess of H_2_) were determined by performing three consecutive experiments at each temperature.

## 4. Conclusions

The effects of H_2_ and temperature on the rates related to the elementary steps of O_2_ adsorption (*k*_1_), desorption (*k*_−1_) and surface bonding (*k*_2_), over a nanosized Au/γ-Al_2_O_3_ catalyst, were investigated by Reversed-Flow Inverse Gas Chromatography. This comparative kinetic study resulted in novel insights in SCO reactant’s sorption processes over Au/γ-Al_2_O_3_.

### 4.1. Effects of Temperature and H_2_ in O_2_ Sorption

H_2_ plays a beneficial role in both the processes of O_2_ adsorption and desorption. For the process of O_2_ surface bonding in the absence of H_2_, there is a “turning point” temperature. While at lower temperatures O_2_ bonding increases slightly, at temperatures higher than 200 °C, the rate of O_2_ surface bonding drastically increases. A similar change in O_2_ desorption, in the absence of H_2_, is noticed: at temperatures higher than 200 °C the rate of O_2_ desorption first stabilizes and at temperatures higher than 250 °C decreases.

The lower O_2_ binding rate at ambient temperatures, which drastically increases at *T* > 200 °C, makes a change in the nature of O_2_ binding over Au/γ-Al_2_O_3_ possible, with rising temperature, in the absence of H_2_. This behavior is in agreement with the general assumption that O_2_ cannot dissociate at low temperatures, but it may be possible on steps at elevated temperatures.

In addition, H_2_ affects to a higher extent the activation energy of O_2_ binding at elevated temperatures, compared to adsorption, desorption and binding at lower temperatures. In this case, H_2_ facilitates stronger O_2_ bonding (from 34.0 to 11.1 kJ·mol^−1^), while low temperature binding remains practically unaffected. This behavior is also consistent with the well-known SCO loss of selectivity at elevated temperatures. The lower energy barriers observed, under H_2_ rich conditions, can be correlated toO_2_ dissociation after hydrogenation.

### 4.2. Comparison of CO and O2 Sorption

Both the processes of CO and O_2_ adsorption are enhanced by the presence of H_2_. However, a drastic increase of the rate of CO adsorption is observed at higher temperatures and in the absence of H_2_.

H_2_ enhances both reactant’s desorption. However, O_2_ desorption is more favored by that of CO, in agreement with the well-known mild bonding of SCO reactant’s at lower temperatures. In the absence of H_2_, and at *T *> 200 °C, the rates of CO as well as O_2_ desorption, from Au/γ-Al_2_O_3_, first stabilize, within the range from 200 to 250 °C, and at even higher temperatures decrease, a behavior related to the well-expected stronger binding of both SCO reactants at high temperatures.

The drastic change in the strength of CO and O_2_ binding on Au/γ-Al_2_O_3_ catalyst is consistent both with well-known high activity of SCO at ambient temperatures as well as with the loss of selectivity, which is observed at higher temperatures.
